# Variable plasmid fitness effects and mobile genetic element dynamics across *Pseudomonas* species

**DOI:** 10.1093/femsec/fix172

**Published:** 2017-12-04

**Authors:** Anastasia Kottara, James P J Hall, Ellie Harrison, Michael A Brockhurst

**Affiliations:** Department of Animal and Plant Sciences, University of Sheffield, Western Bank, Sheffield, S10 2TN, UK

**Keywords:** horizontal gene transfer, mobile genetic elements, conjugative plasmids, bacteria-plasmid coevolution, experimental evolution

## Abstract

Mobile genetic elements (MGE) such as plasmids and transposons mobilise genes within and between species, playing a crucial role in bacterial evolution via horizontal gene transfer (HGT). Currently, we lack data on variation in MGE dynamics across bacterial host species. We tracked the dynamics of a large conjugative plasmid, pQBR103, and its Tn5042 mercury resistance transposon, in five diverse *Pseudomonas* species in environments with and without mercury selection. Plasmid fitness effects and stability varied extensively between host species and environments, as did the propensity for chromosomal capture of the Tn5042 mercury resistance transposon associated with loss of the plasmid. Whereas *Pseudomonas fluorescens* and *Pseudomonas savastanoi* stably maintained the plasmid in both environments, the plasmid was highly unstable in *Pseudomonas aeruginosa* and *Pseudomonas putida*, where plasmid-free genotypes with Tn5042 captured to the chromosome invaded to higher frequency under mercury selection. These data confirm that plasmid stability is dependent upon the specific genetic interaction of the plasmid and host chromosome rather than being a property of plasmids alone, and moreover imply that MGE dynamics in diverse natural communities are likely to be complex and driven by a subset of species capable of stably maintaining plasmids that would then act as hubs of HGT.

## Teaser

Bacteria share genes via mobile genetic elements but the dynamics of these vary extensively between species.

## INTRODUCTION

Bacterial evolutionary innovation and adaptation is often dependent upon the acquisition of novel accessory genes carried on mobile genetic elements (MGE) (Frost *et al.*^2005^). This fundamental evolutionary process is termed horizontal gene transfer (HGT) (Thomas and Nielsen 2005). Conjugative plasmids are important vectors of HGT as they can be both inherited vertically during cell division and transmitted horizontally by conjugation within and between bacterial species (Norman, Hansen and Sørensen 2009). In addition to genes for their own replication, propagation and stability, many plasmids also encode a complement of accessory genes: bacterial genes that do not benefit the plasmid directly but can be beneficial for the bacterial host under specific environmental conditions, e.g. traits such as antibiotic and metal resistance (Eberhard 1990). Understanding the maintenance and spread of accessory genes is a pressing concern for microbiologists, particularly because of the grave threat that plasmid-borne antibiotic resistance poses in opportunistic infection (zur Wiesch *et al.*^2011^; Holmes *et al.*^2016^).

The fate of a plasmid within a host is determined by several key factors: the cost of plasmid carriage, the conjugation rate and segregation rate of the plasmid, and the strength of positive selection on plasmid-borne accessory genes that will vary according to the environment (Simonsen 1991; Bergstrom, Lipsitch and Levin 2000; Slater *et al.*^2008^). In environments where the benefits of accessory genes outweigh the costs of carrying the plasmid (i.e. where the plasmid–bacteria interaction is mutualistic) plasmids may be maintained at high frequency through positive selection (San Millan *et al.*^2014^; Harrison *et al.*^2015^). However, over longer evolutionary timescales, it is likely that positive selection will favour the integration of the beneficial accessory genes into the host chromosome (Bergstrom, Lipsitch and Levin 2000), a process facilitated by accessory genes often being located on transposons or other integrative elements (Osborn and Böltner 2002). In contrast, in environments where the cost to the host of carrying the plasmid outweighs the benefit of the plasmid-borne accessory genes (i.e. when the bacteria–plasmid interaction is parasitic), plasmids will be lost due to purifying selection unless the rate of loss is counteracted by a sufficiently high rate of conjugation such that they are maintained by infectious transmission (Bergstrom, Lipsitch and Levin 2000; Hall *et al.*^2016^). These key parameters affecting plasmid population dynamics are likely to vary, leading to differences in the dynamics of plasmids and their constituent MGEs among host species.

Variation in plasmid population dynamics between host species has typically been considered in terms of plasmid host range, i.e. the subset of host species wherein the plasmid can be stably maintained (Bahl, Hansen and Sørensen 2009). However, the ability to infect and replicate may not reflect the long-term stability of a plasmid in a host population, because of high rates of segregation and/or plasmid costs (Turner, Cooper and Lenski 1998). Previous studies have demonstrated variation in long-term plasmid population dynamics in different hosts (De Gelder *et al.*^2007^; Sota *et al.*^2010^; Porse *et al.*^2016^) but have tended to focus on one selective environment and do not assess the role of chromosomal capture of accessory genes.

Here, we quantify for a range of bacterial host species the fitness effects of plasmid acquisition, and variation in the plasmid population dynamics, under both negative selection (plasmid is parasitic) and positive selection (plasmid is mutualistic). Our experimental system consisted of a conjugative plasmid, pQBR103, conferring mercury resistance via a *mer* operon encoded on a Tn5042 transposon, and five *Pseudomonas* species representing *Pseudomonas fluorescens*, *Pseudomonas putida*, *Pseudomonas savastanoi*, *Pseudomonas aeruginosa* and *Pseudomonas stutzeri.* Populations were propagated with and without mercury and the dynamics of the mercury resistance phenotype, the pQBR103 plasmid and the Tn5042 transposon were tracked over time. We report that the fitness effects of plasmid carriage and MGE dynamics varied extensively between the different *Pseudomonas* species, indicating that plasmid-host dynamics are governed by species-specific interactions between plasmids and the host chromosome.

## MATERIALS AND METHODS

### Bacterial strains and culture conditions

We utilised five phylogenetically diverse *Pseudomonas* species (Fig. S1, Supporting Information) isolated from a range of environments: *P. fluorescens* SBW25 was isolated from the leaf surface of the sugar beet plant (Rainey, Bailey and Thompson 1994); *P. putida* KT2440 is a derivative of the toluene degrading and soil isolate *P. putida* mt-2 (Nakazawa and Yokota 1973); *P. savastanoi pv. phaseolicola* 1448A is a plant-associated isolate and pathogen of the common bean (Arnold *et al.*^2011^); *P. stutzeri* JM300 (DSM10701) is a denitrifying soil isolate (Busquets *et al.*^2012^); *P. aeruginosa* PAO1 is a derivative of the original Australian PAO that was isolated from a wound in Alfred hospital in Melbourne (Holloway 1955). Furthermore, *P. savastanoi pv. phaseolicola* carries two native plasmids (131 and 51 kb) (Joardar *et al.*^2005^), while the other aforementioned *Pseudomonas* species are plasmid-free isolates. The plasmid used in this study, pQBR103, was isolated from the natural bacterial community colonising the rhizosphere and phyllosphere of sugar beets (Lilley *et al.*^1996^; Tett *et al.*^2007^). *Pseudomonas* species were labelled by directed insertion of a gentamicin resistance (Gm^R^) marker using the mini-Tn7 transposon system (Lambertsen, Sternberg and Molin 2004). Plasmid-carrying Gm^R^ strains were obtained by incubating streptomycin resistant (Sm^R^) *P. fluorescens* SBW25 stocks that were carrying pQBR103 plasmid, with the plasmid-free Gm^R^ strains for 48 h and spreading on KB agar plates containing 10 μg mL^−1^ gentamicin and 20 μM of mercury (II) chloride to select for transconjugant colonies (Simonsen *et al.*^1990^). All experiments were conducted in 6 mL King's B growth medium in 30 mL universal vials (microcosms) at 28°C in shaking conditions (180 rpm).

### Competitive fitness assay

Six individual colonies of each *Pseudomonas* species containing the ancestral plasmid were grown overnight in microcosms and later each was competed against the relevant isogenic plasmid-free strain across a range of mercury (II) chloride concentrations from 0 to 60 μΜ. Relative fitness was measured by mixing differentially labelled test (plasmid-bearer, labelled gentamicin) and reference (plasmid-free, wild-type) in ∼1:1 ratio, diluted 100-fold in KB microcosms containing the relevant mercury concentration and incubated at 28°C for 48 h. Samples were plated on KB agar plates at the beginning and end of the competition and replica plated onto KB agar plates supplemented with selective concentration of gentamicin to estimate the density of plasmid bearers. The relative fitness was calculated as the selection rate (*r*) (Lenski *et al.*^1991^) and normalised for the marker effects by subtracting the selection rate of gentamicin labelled, plasmid-free strains over the plasmid-free, wild-type strains.

### Evolution experiment

Prior to the evolution experiment 12 individual colonies of each *Pseudomonas* species carrying the ancestral plasmid were reconditioned from frozen stocks overnight in KB 6 mL microcosms at 28°C with shaking (180 rpm/min), after which time 1% of each population was transferred to grow for 24 h in fresh KB microcosms containing 50 μΜ of mercury (II) chloride at the same temperature and shaking conditions. This step was necessary to ensure high starting frequencies of plasmid carriage across all the tested bacterial strains due to the high segregation rate of the plasmid in some host strains (e.g. *P. putida* KT2440, data not shown).

For the evolution experiment, six clonal populations of each bacterial strain were grown in absence of mercury and six populations were grown in the presence of mercury selection (50 μΜ of mercury (II) chloride) in KB microcosms at 28°C with shaking (180 rpm/min). Every 48 h 1% of each population was transferred into a fresh microcosm for 60 transfers [∼400 generations]. The density of each bacterial population was monitored every four transfers by plating a sample onto KB agar plates incubated at 28°C for 48 h. Each plate was then replica plated onto KB agar plates supplemented with 100 μΜ of mercury (II) chloride to assess the frequency of mercury resistance. Subsequently, 24 bacterial colonies were randomly selected from each mercury replica plate. The prevalence of the mercury resistance transposon, Tn5042, and the plasmid, pQBR103, was estimated at transfers 2, 4 and 8 and then at every 12 transfers by PCR screening of the 24 bacterial colonies randomly selected from each clonal population. The PCR screening was designed as previously described (Harrison *et al.*^2015^) with two sets of primers, one targeted to *mer* operon on Tn5042 transposon [forward primer-TGCAAGACACCCCCTATTGGAC, reverse primer-TTCGGCGACCAGCTTGATGAAC] and the other to origin of replication of the plasmid (*ori*V) [forward primer-TGCCTAATCGTGTGTAATGTC, reverse primer-ACTCTGGCCTGCAAGTTTC]. Detection limits were estimated by Poisson calculations.

### Statistical analyses

Statistical analyses were performed using RStudio version 3.2.3 (R Core Team 2013). We used a linear model to analyse the variation in the plasmid cost between the *Pseudomonas* species and mercury selection environments where mercury was fitted as a quadratic term for each species [*r* ∼ species background × mercury × mercury^2^]. Mercury was fitted as a quadratic term as 4/5 species showed non-linear fitness responses to the increase of mercury. We further used model comparisons to test the linearity of genotype by environment interactions. To analyse the end-point frequency of mercury resistance and plasmid prevalence of the evolution experiment, we fitted a linear model to the different *Pseudomonas* species across the mercury selection environments (0 μM and 50 μM mercury (II) chloride). Plasmid population dynamics in the *P. stutzeri* parallel evolving populations were further investigated by comparing plasmid prevalence across mercury conditions using a one way-ANOVA. Plasmid prevalence was estimated as the area under the curve using the function *auc* of the package ‘flux’ (Jurasinski, Koebsch and Hagemann 2012).

## RESULTS

### Plasmid fitness costs varied between *Pseudomonas* species

**Figure 1.**
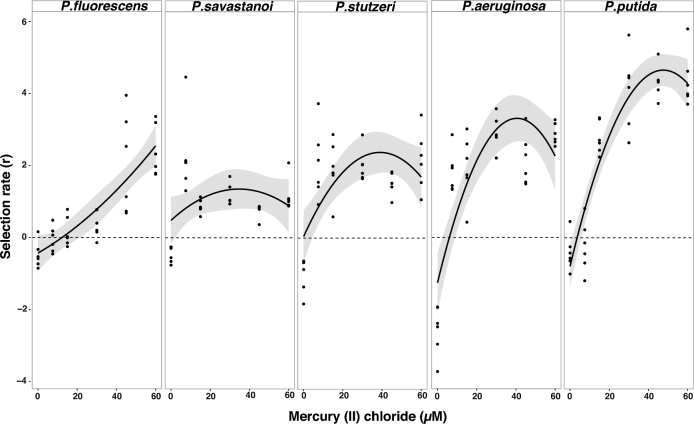
Fitness of *Pseudomonas* species carrying the plasmid measured as selection rate in a mercury regime ranging from 0–60 μΜ mercury (II) chloride. Solid circle (•): each clonal population carrying the plasmid and competing with the isogenic wild-type strain; solid line (**-**): curve fitting mercury as a quadratic term for each species; dashed line (**–**): selection rate 0 indicates no difference between test and reference strains; shaded area: confidence interval of fitting curve.

We first quantified the fitness effect of plasmid carriage on bacterial hosts using competition experiments across a wide range of mercury environments, ranging from no mercury where the plasmid confers no benefit (0 μM mercury (II) chloride) to high levels of mercury contamination where the plasmid and its mercury transposon are essential (60 μM mercury (II) chloride). Although, in general, the plasmid was costly to host species in the absence of mercury, the magnitude of the cost and the form of the fitness response with increasing mercury concentration varied between species (effect of species background x mercury x mercury^2^ interaction, ANOVA F_4,159_ = 9.616, *P* = 5.451e−07; Fig. 1). For instance, in the absence of mercury the plasmid was highly costly in *P. aeruginosa*, whereas it imposed a far lower fitness cost in *P. fluorescens* and *P. savastanoi.* Moreover, whereas *P. fluorescens* showed a positive and linear fitness response with increasing mercury concentration (effect of mercury (*P. fluorescens* fitness data), ANOVA F_1,32_ = 72.829, *P* = 9.425e−10), the fitness of the other plasmid bearing *Pseudomonas* species increased rapidly at low concentrations of mercury (>/ = 7.5 μM mercury (II) chloride) (species background by mercury^2^ interaction, ANOVA F_4,159_ = 10.34, *P* = 1.809e−07). Model comparison revealed that the fitness response to mercury concentration was non-linear in 4/5 of the species (ANOVA F = 19.058, *P* < 2.2e−16). Furthermore, minimum inhibitory concentration assays showed that species varied both in their inherent susceptibility to mercury (Fig. S2, Supporting Information) and in the level of mercury resistance conferred by pQBR103 (Fig. S3, Supporting Information). These data demonstrate extensive variation in the fitness effect of plasmid acquisition across the *Pseudomonas* phylogeny, suggesting that even in relatively closely related bacteria (Fig. S1, Supporting Information), plasmids and their accessory genes can have markedly different fitness effects.

### Variable dynamics of mercury resistance over time

**Figure 2.**
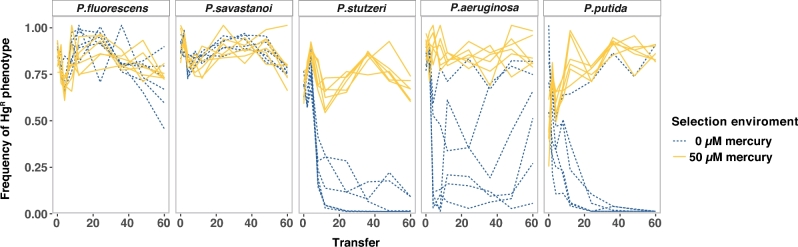
Frequency of mercury resistance in the parallel evolving clonal populations in absence (blue dashed lines) and presence (50 μM) of mercury (yellow lines) throughout the selection experiment. Each line represents a clonal population evolving during the evolution experiment.

To examine the consequences of these fitness effects on long-term population dynamics, we tracked mercury resistance in populations evolved for ∼400 generations either with or without mercury selection. Mercury resistance was assessed by replica plating populations every four transfers onto mercury selective media. At the end of the experiment, we found that the level of mercury resistance varied between *Pseudomonas* host species and with mercury environment (species by mercury interaction, ANOVA F_4,50_ = 8.808, *P* = 1.83e−05). As expected, mercury selection promoted the maintenance of mercury resistance in all host species. Without mercury selection, the maintenance of mercury resistance was highly dependent on host species (Fig. 2). In the absence of mercury, resistance was maintained throughout the experiment at high frequency in *P. fluorescens* and *P. savastanoi*, but lost rapidly from *P. putida* and *P. stutzeri.* Specifically, mercury resistance was not detected in 5/6 and 4/6 replicates of *P. putida* and *P. stutzeri*, respectively, at the end of the experiment (>95% probability of detecting HgR if present at frequencies of ≥9.1%). In *P. aeruginosa*, resistance dynamics varied across replicate populations, with final frequencies ranging from 4% to 80% of the population evolving in mercury-free environment (>95% probability of detecting HgR if present at frequencies of ≥3.2%).

### Variation in plasmid and transposon dynamics over time

**Figure 3.**
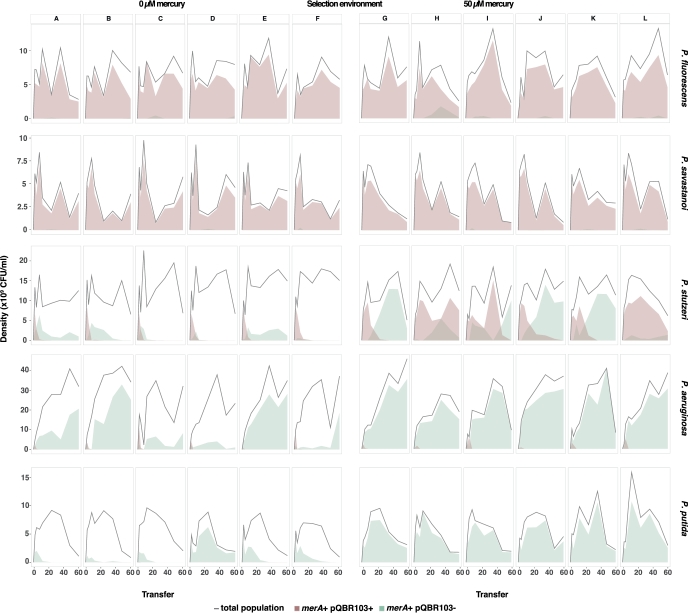
Population density and tracked mercury resistance transposon, Tn5042 and plasmid density. (**A**–**F**) clonal populations evolving in absence of mercury; (**G**–**L**) clonal population evolving in presence of 50 μΜ mercury. Population density (grey line); tracked mercury transposon in presence of the plasmid (brown density plot area); tracked mercury transposon when the plasmid was not detected (green density plot area).

Plasmid-borne accessory genes can transfer to the chromosome, allowing loss of a redundant plasmid but the retention of the resistance gene(s) (Bergstrom, Lipsitch and Levin 2000; Hall *et al.*^2016^). Therefore, we used PCR to test whether mercury resistant clones isolated during the experiment still carried the plasmid, or whether it had been lost following acquisition of chromosomal mercury resistance. Plasmid maintenance varied between the different *Pseudomonas* host species (effect of species background, ANOVA F_4,50_ = 158.33, *P* < 2e−16; Fig. 3). The plasmid was maintained in *P. fluorescens* and *P. savastanoi* in both mercury environments: although pQBR103-free mercury resistant clones arose in several populations, they did not invade over the course of the experiment. *Pseudomonas stutzeri* maintained the plasmid at intermediate levels, but only under mercury selection, and even then the plasmid was lost in 3/6 populations due to invasion of plasmid-free mercury resistant clones (plasmid prevalence by mercury environment in *P. stutzeri*, ANOVA F_1,10_ = 12.86, *P* = 0.004). In contrast, we observed rapid, complete loss of the plasmid in *P. putida* and *P. aeruginosa* regardless of mercury selection (>95% probability of detecting pQBR103 if present at frequencies ≥2.1%), and where mercury resistance was observed in these hosts this resulted from chromosomal capture of the resistance genes (Fig. 3). These findings indicate clear variation in plasmid stability between hosts dependent upon the environment, and moreover, variation between host species in the propensity to replace plasmid-borne resistance with chromosomal resistance via capture of Tn5042.

## DISCUSSION

It is clear that the relationship between a plasmid and its host is highly context dependent (De Gelder *et al.*^2007^; Humphrey *et al.*^2012^). Here, the same plasmid can levy different costs on different hosts: we found that the cost of plasmid carriage was 2.5-fold higher in *P. aeruginosa* compared with other *Pseudomonas* species. The environment can convert the relationship from parasitism to mutualism by selection for plasmid-borne genes: in our case application of 7.5 μM mercury was sufficient for pQBR103 to go from a significant parasite (inhibiting the growth of *P. aeruginosa* by 13% compared with plasmid-free) to a clear mutualist (enhancing the relative fitness of its host by 71%), similar to previous work (Gullberg *et al.*^2014^; Hall *et al.*^2015^). Furthermore, the effect of environmental selection varies between hosts, implying that the addition of mercury to the environment does not benefit plasmid bearers of all species equally. This is clearest in the case of *P. fluorescens*, which, compared with the other species, required higher levels of mercury selection for the plasmid to be beneficial. A potential limitation of our fitness measurements is that the rapid segregational loss of the plasmid observed in some of the host species, most notably *P. putida*, may have led to the underestimation of plasmid costs in these hosts. Nevertheless, the fact that the emergence and success of such segregants varies between species is consistent with our main finding. Previously, we showed that different mercury resistance plasmids imposed varying costs, which varied for each plasmid depending on the selective environment, despite the fact that the plasmids shared the same resistance genes (Hall *et al.*^2015^). Together, the data paint an increasingly complex picture whereby plasmids, hosts and the environment interact to determine whether plasmid-bearers suffer from the burden of carriage or profit from the accessory genes that plasmids often provide. This is consistent with a recent meta-analysis of variation in plasmid costs, which showed that the variation in costs for a given plasmid across different host genotypes can be as large as the variation for different plasmids in a given host (Vogwill and MacLean 2015).

Consistent with the short-term measurements of fitness, we observed divergent long-term dynamics between the different species. *Pseudomonas fluorescens* and *P. savastanoi* maintained the plasmid, while *P. aeruginosa* and *P. putida* lost the plasmid, regardless of mercury selection. *Pseudomonas stutzeri* plasmid maintenance required mercury selection. The extinction of pQBR103 in *P. aeruginosa* populations can be readily explained by the high cost the plasmid levies on this host (in the absence of mercury, average plasmid prevalence was decreased 42% within the first two transfers and 96% from transfer 2 to 4), but measurements of fitness can only partly explain the long-term dynamics. For example, long-term plasmid population dynamics in *P. fluorescens* and *P. putida* were widely divergent despite the fact that costs-of-carriage were similar. Rates of segregation and/or conjugation, which are known to vary between species (Hall *et al.*^2016^), may help explain longer term maintenance. Alternatively, species may vary in their ability to accommodate an incoming plasmid through compensatory mutation. Plasmid cost is likely to come primarily from specific interactions between plasmid and host (Baltrus 2013; San Millan *et al.*^2014^): for some hosts, modulating such interactions may be readily achieved through mutation, for others it may be easier to simply lose the plasmid. The cost of pQBR103 carriage by *P. fluorescens*, for example, has been shown to be associated with rapid compensatory evolution, facilitated by mutations targeting the *gac*S/A system (Harrison *et al.*^2015^). Though the *gacS/A* system is a conserved global regulatory system, it responds to different signals and controls different processes in each species, and may be tightly associated with niche occupation (Lapouge *et al.*^2008^). In other hosts, mutations to *gacA/S* might not alleviate plasmid costs, may impose excessively negative pleiotropic effects or might not occur readily enough, all of which would limit plasmid survival (Harrison *et al.*^2016^).

Theory predicts that under selection, beneficial plasmid accessory genes are captured by the chromosome, and the plasmid is lost (Bergstrom, Lipsitch and Levin 2000). In all of the species, we tested we detected mutants that had lost the plasmid but maintained mercury resistance, presumably through chromosomal acquisition of the mercuric reductase MerA. Acquisition of MerA most likely occurred by the transposition of the Tn5042 mercury resistance transposon from the plasmid onto the chromosome: Tn5042 is wide spread in the environment and across pQBR plasmids (Mindlin *et al.*^2005^; Hall *et al.*^2015^) and is known to mobilise to the chromosome (at least in *P. fluorescens*; Harrison *et al.*^2015^). Studies have extensively reported that resistance genes are frequently located on transposons, increasing the propagation and persistence of the resistance genes in the environment (Frost *et al.*^2005^; Partridge *et al.*^2009^). Interestingly, we found that genotypes carrying chromosomal mercury resistance did not only invade lineages treated with mercury, but also formed a substantial fraction of the *P. aeruginosa* and *P. putida* populations evolved without mercury. These data suggest that the spread of resistance could be a species-specific characteristic and that for some species even transient plasmid carriage is sufficient for successful mobilisation of resistance genes to the chromosome and subsequent spread. Species that are poor plasmid hosts need not be excluded from the mobile gene pool, provided they can transfer plasmid-borne accessory genes to their chromosome rapidly enough.

Our work has shown that long-term plasmid-host dynamics vary with environmental selection and host genotype. Laboratory experiments such as these are revealing a complex and contingent partnership but are necessarily simple, stripping away many of the details of the natural environment. Natural environments are likely to be spatially structured and heterogeneous, which could act to both promote and impede HGT between species; spatially structured environments can potentially impede encounters between cells, reducing opportunities for HGT, whereas, in such environments, biofilm growth predominates and environmental heterogeneity can promote species coexistence, which could increase opportunities for interspecific HGT (Heuer and Smalla 2012). In the wild, plasmids and their hosts do not exist in a dyadic relationship—microbial communities contain many species (Lozupone and Knight 2007) and many different mobile elements (Norman, Hansen and Sørensen 2009) and a small subset of hosts able to maintain plasmids could act as hubs of exchange, spreading genes throughout the community (Hall *et al.*^2016^). In this study, we found that two out of five of the *Pseudomonads* tested maintained the plasmid, indicating that hubs may be fairly common in this genus, although further work is required to identify the genetic basis of plasmid maintenance across the species tested. In addition, our data suggest that selection for plasmid-borne genes may not be a widespread mechanism for maintenance, since plasmid fate was only enhanced by mercury treatment for one of the species we tested (*P. stutzeri*). If maintenance and spread of plasmids is governed more by species presence than by selection for plasmid-borne genes, this has important implications for the control of resistance elements: without an understanding of the species involved, attempts to limit the spread of resistance by limiting antibiotic use (for example) may prove to be unsuccessful.

## SUPPLEMENTARY DATA

Supplementary data are available at *FEMSEC* online.

Supplementary material Supplementary data are available at *FEMSEC* online.
