# Supplementary material for: Variable plasmid fitness effects and mobile genetic element dynamics across Pseudomonas species
Source: FEMS Microbiol Ecol. 2017 Dec 4;94(1):fix172. doi: 10.1093/femsec/fix172 (PMC5812508; doi:10.1093/femsec/fix172)
Supplement: Supplementary material — Supplementary data are available at FEMSEC online. [file fix172_supp.docx]

**Supplementary Information**

**Figure S1.** Phylogenetic tree of *Pseudomonas* species used in this study. The tree was build using the whole genome sequence data in the online pipeline REALPHY 1.10 (Bertels *et al*. 2014). The genome sequence of *P. fluorescens* SBW25 was used as a reference genome where the other *Pseudomonas* genomes were aligned by using bowtie2 (Langmead and Salzberg 2012). PhyML was used to build the maximum likelihood tree; the tree topologies were obtained based on the nearest-neighbor interchanges (NINs) (Guindon *et al*. 2010). FigTree v1.4.3 was used to display and draw the tree (http://tree.bio.ed.ac.uk/software/figtree/). Branch labels: relative distances along the branches.

**Figure S2.** Growth of plasmid-free *Pseudomonas* species in mercury environment ranging from 0-60 μΜ mercury(II)chloride. Four individual colonies of each *Pseudomonas* species were grown at the exponential phase (optical density at 600 nm: ~0.4) and later each diluted 1000-fold in KB containing the relevant mercury concentration and incubated at 28°C for 48 h. Points represent the mean growth (n=4) measured as optical density at 48 h, based on the absorbance at 600 nm. Error bars represent SEM of four clonal replicates.

**Figure S3.** Growth of plasmid-bearing *Pseudomonas* species in mercury environment ranging from 0-135 μΜ mercury(II)chloride at 48 h. Four individual colonies of each *Pseudomonas* species containing the ancestral plasmid were grown at the exponential phase (optical density at 600 nm: ~0.4) and later each diluted 1000-fold in KB containing the relevant mercury concentration and incubated at 28°C for 48 h. Points represent the mean growth (n=4) measured as optical density at 48 h, based on the absorbance at 600 nm. Error bars represent SEM of four clonal replicates.
